# Recent advances in renal imaging

**DOI:** 10.12688/f1000research.16188.1

**Published:** 2018-11-29

**Authors:** Joshua M. Thurman, Faikah Gueler

**Affiliations:** 1 Department of Medicine, University of Colorado School of Medicine, Aurora, CO, USA; 2 Department of Nephrology, Hannover Medical School, Hannover, Germany

**Keywords:** Kidney, imaging, diagnosis, inflammation, fibrosis

## Abstract

Kidney diseases can be caused by a wide range of genetic, hemodynamic, toxic, infectious, and autoimmune factors. The diagnosis of kidney disease usually involves the biochemical analysis of serum and blood, but these tests are often insufficiently sensitive or specific to make a definitive diagnosis. Although radiologic imaging currently has a limited role in the evaluation of most kidney diseases, several new imaging methods hold great promise for improving our ability to non-invasively detect structural, functional, and molecular changes within the kidney. New methods, such as dynamic contrast-enhanced magnetic resonance imaging (DCE-MRI) and blood oxygen level-dependent (BOLD) MRI, allow functional imaging of the kidney. The use of novel contrast agents, such as microbubbles and nanoparticles, allows the detection of specific molecules in the kidney. These methods could greatly advance our ability to diagnose disease and also to safely monitor patients over time. This could improve the care of individual patients, and it could also facilitate the evaluation of new treatment strategies.

## Introduction

Kidneys have numerous physiologic functions beyond the clearance of uremic toxins. They maintain the balance of electrolytes and water, help maintain acid–base balance, produce erythropoietin, and are critical for bone and mineral metabolism. To carry out these diverse functions, kidneys have many unique anatomic and ultrastructural features. The various functions also require different specialized cells to detect the composition of body fluids and to respond to physiologic changes. Unique cell types within the glomerulus and along the renal tubules restrict the passage of some molecules while mediating the transport of others into and out of the urine.

Because of this broad range of functions, the kidney can be affected by many different genetic, hemodynamic, toxic, infectious, and autoimmune insults. Kidney disorders are usually diagnosed by biochemical measurements of serum and blood, but these tests are often insufficiently sensitive or specific to make a definitive diagnosis. Measuring serum creatinine, for example, is the most common method of detecting a reduction in glomerular filtration, but it is an insensitive marker of kidney function and it does not discriminate between the different causes of kidney injury. Anatomic changes can also be difficult to evaluate. In patients with renal artery stenosis, for example, similar degrees of vascular obstruction seen on ultrasound or angiography can have very different functional consequences. New methods for detecting molecular, anatomic, and functional changes within the kidney would therefore improve our ability to diagnose many different diseases.

Radiologic imaging currently has only a limited role in the diagnosis of kidney disease. Imaging is primarily used for diagnosing nephrolithiasis or gross anatomic defects, such as cystic disease, malignancies, and obstructive nephropathy. For most other kidney diseases, imaging is used simply to assess kidney size and density, crude markers of parenchymal damage ^1^. Sophisticated new radiologic methods have been developed in recent years that enable functional measurement of physiologic processes and quantitative assessment of molecular markers within tissues. Contrast agents are substances that can enhance the radiographic visibility of structures, such as the vasculature. Imaging probes, on the other hand, are agents that are used to detect specific biologic processes or molecules ^2^, and new probes have been developed to detect and quantify specific changes in the composition or function of the kidneys. These new modalities and tools have the potential to improve our ability to diagnose disease, detect changes in kidney structure and composition, and non-invasively monitor a patient’s response to treatment.

## Anatomic imaging

A fundamental use of radiologic studies is to examine the basic structure of an organ. Ultrasound is frequently used to measure kidney size, search for the presence of renal masses or cysts, detect kidney stones, and determine whether there is urinary obstruction ^3^. Doppler imaging can be added to examine flow in the renal arteries and veins or to measure the resistive index. Computed tomography (CT) and magnetic resonance imaging (MRI) are also frequently used for these purposes. However, the use of potentially toxic contrast media limits the application to patients with an estimated glomerular filtration rate (eGFR) of <30 ml/minute. For CT scans, iodinated contrast has been linked to nephrotoxicity. It is possible that the risks are not as high as previously thought, however, and this is an area of ongoing research ^4, 5^. For MRI, the use of gadolinium-based contrast agents (GBCAs) in the past has been linked to nephrogenic systemic fibrosis (NSF), especially in patients with acute kidney injury (AKI), chronic kidney disease (CKD), and end-stage renal disease (ESRD) ^6^. However, newer macrocyclic GBCAs such as gadobenate dimeglumine or gadobutrol have not been linked to NSF and have been used safely in patients with stage 3–5 CKD in a prospective clinical study ^7– 9^.

**Figure 1.**
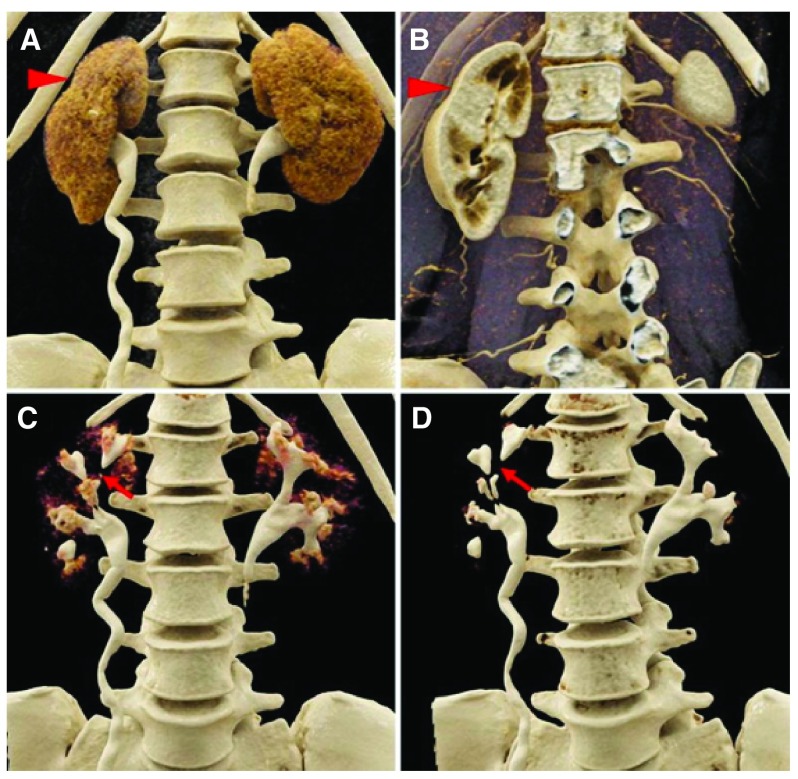
Cinematic rendering of a computed tomography image of a scarred kidney. Coronal, contrast-enhanced computed radiography visualizations of a 42-year-old woman with a history of recurrent urinary tract infections. With soft tissue window settings in the **A**) excretory/urographic and **B**) arterial phases, contour abnormalities in the right kidney that correspond to regions of scarring are apparent (red arrowheads). **C**, **D**) During the excretory/urographic phase of this study, the windowing can be adjusted to optimize the visualization of the collecting systems and ureters. In the right kidney, note the extensive irregularities of the collecting system, including multiple calyceal strictures (red arrows). Reproduced with permission from Rowe *et al.* ^14^.

Improved methods of acquiring and analyzing anatomic data will improve the accuracy with which lesions can be detected. This could be important for diseases like polycystic kidney disease (PKD), where radiologic measurements of cyst and kidney volume have been closely linked with a decline in kidney function ^10^. New analytic methods, such as convolutional neural networks, may increase the speed and accuracy of volume measurements in PKD ^11^. Another method of image reconstruction, referred to as cinematic rendering, uses algorithms to add light and shading to CT images and make them appear more realistic ^12, 13^. Although this method may make it easier to identify and evaluate intra-renal lesions ^14^ ( Figure 1), more experience at additional centers is necessary in order to determine its usefulness.

## Imaging of renal perfusion and glomerular filtration rate

Renal blood flow (RBF) is frequently reduced in patients with ischemic AKI, renal artery stenosis, obstructive nephropathy, or decreased mean arterial blood pressure. Changes in RBF can affect the whole organ, but arteriovenous fistulas (AVFs) or vascular thrombosis can affect blood flow focally. Major vascular occlusions and larger AVFs can usually be detected by Doppler ultrasound, but it cannot resolve regional blood flow or determine whether blood flow is sufficient for the kidney’s metabolic needs. Several functional imaging methods are being developed to more accurately quantify RBF into the kidney, assess the adequacy of blood flow based on functional measurements, and measure the rate at which plasma is filtered by the kidney (GFR).

### Renal perfusion

**Figure 2.**
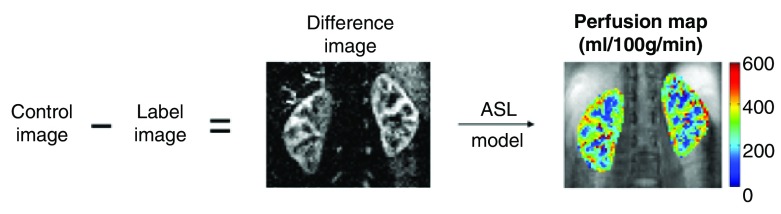
Overview of arterial spin labeling (ASL). A control image is obtained without contrast. A pulse is then used to magnetize the blood, and a delay allows the blood to enter the kidney. The labeled image is then subtracted from the baseline image, generating a map of the signal difference created by the perfused blood. In healthy volunteers, the amount of signal due to inflowing blood is in the order of 5% of the non-background suppressed baseline tissue magnetization. Reproduced with permission from Nery, Gordon, and Thomas ^16^.

Arterial spin labeling (ASL) is an MRI method that allows the assessment of blood flow throughout the entire kidney. ASL utilizes magnetically tagged water as an intrinsic contrast agent, so it is safe in patients with renal insufficiency ^15^. The basic principle of ASL is that a static image of an organ is generated. Blood water is then magnetically tagged with a radiofrequency (RF) pulse before it enters the tissue of interest, and then another image is created in which the magnetization of the inflowing blood is also captured. Subtraction of labeled images from the control images eliminates static tissue signal, and the remaining signal is a relative measure of perfusion proportional to RBF ( Figure 2) ^16^. Although the resolution of this method was initially quite poor, significant improvements in imaging acquisition techniques have been made which now enable the discrimination of values in the cortex and medulla. The PARENCHIMA network recently reviewed 53 studies on renal perfusion by ASL in patients with various forms of nephropathy ^17^. Although there is no gold standard against which to validate ASL, the authors concluded that the method has good reproducibility.

**Figure 3.**
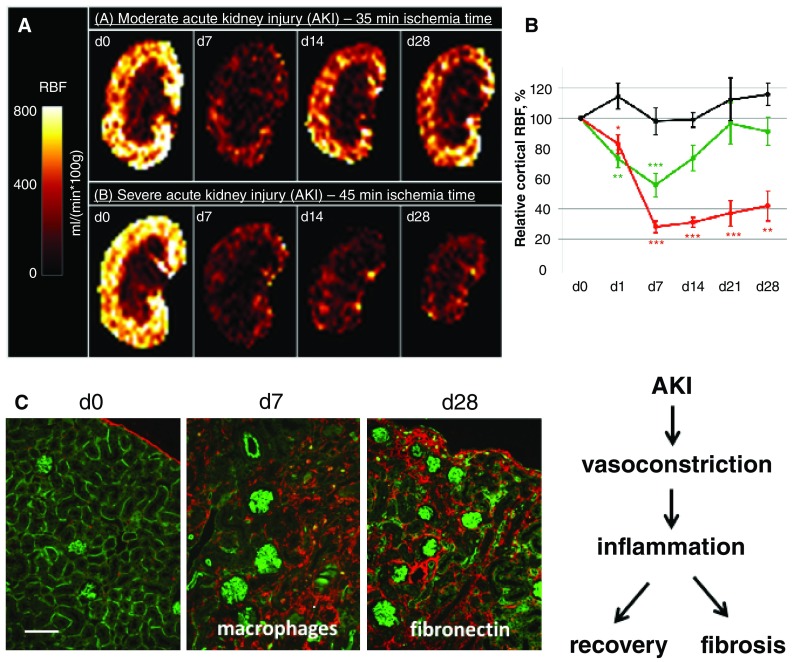
Arterial spin labeling (ASL) in a model of acute kidney injury (AKI). **A**) ASL in a mouse model of transient AKI induced by short-term ischemia/reperfusion injury (IRI; 35 minute clipping of the renal pedicle, green line, upper row) compared to prolonged ischemia time (45 minute clipping and resulting in renal fibrosis, red line, lower row). **B**) Quantification over time shows the permanent impairment of renal cortical blood flow due to prolonged ischemia time causing renal fibrosis. The black line represents the contralateral not clipped kidney. Reproduced with permission from Hueper *et al*. ^19^. **C**) Histology (45 minute IRI) shows lectin-perfused kidneys where patent capillaries are stained in light green in the healthy kidney (d0 double stained in red with fibronectin), at d7 macrophages (red) are infiltrated in area with loss of patent capillaries, and at d28 hypoperfused areas show enhanced scarring with fibronectin expression (red; bar represents 100 µm). RBF, renal blood flow.

Ischemia reperfusion injury (IRI) is a common cause of AKI and has a high incidence after major cardiac surgery or solid organ transplantation. For cardiac surgeries in which cardiopulmonary bypass is needed, the likelihood of AKI may be as high as 30% ^18^. ASL offers an excellent method of detecting early renal damage, since ischemia causes vascular constriction and a reduction of RBF. Much work has been done in standardized mouse and rat models to correlate reductions in RBF (as assessed by ASL) with histologic changes at different time points after AKI onset ( Figure 3) ^19^. Reductions in RBF also correlate with AKI severity ^20^ and with long-term outcomes ^21^. Initially, there is a functional reduction in RBF, and this worsens over the first week because of increasing leukocyte infiltration and inflammation of the kidneys. Then, either recovery starts or ongoing inflammation leads to a permanent reduction of RBF and scarring of the kidneys. A pilot study in human patients showed that RBF is lower in patients with AKI than in control patients ^22^. In kidney transplantation, the correlate of AKI is delayed allograft function (DGF). Studies in transplant patients have shown that RBF is reduced in patients with DGF compared to those with functioning allografts ^23, 24^. Lower RBF is also predictive of graft failure in patients with DGF ^24^.

**Figure 4.**
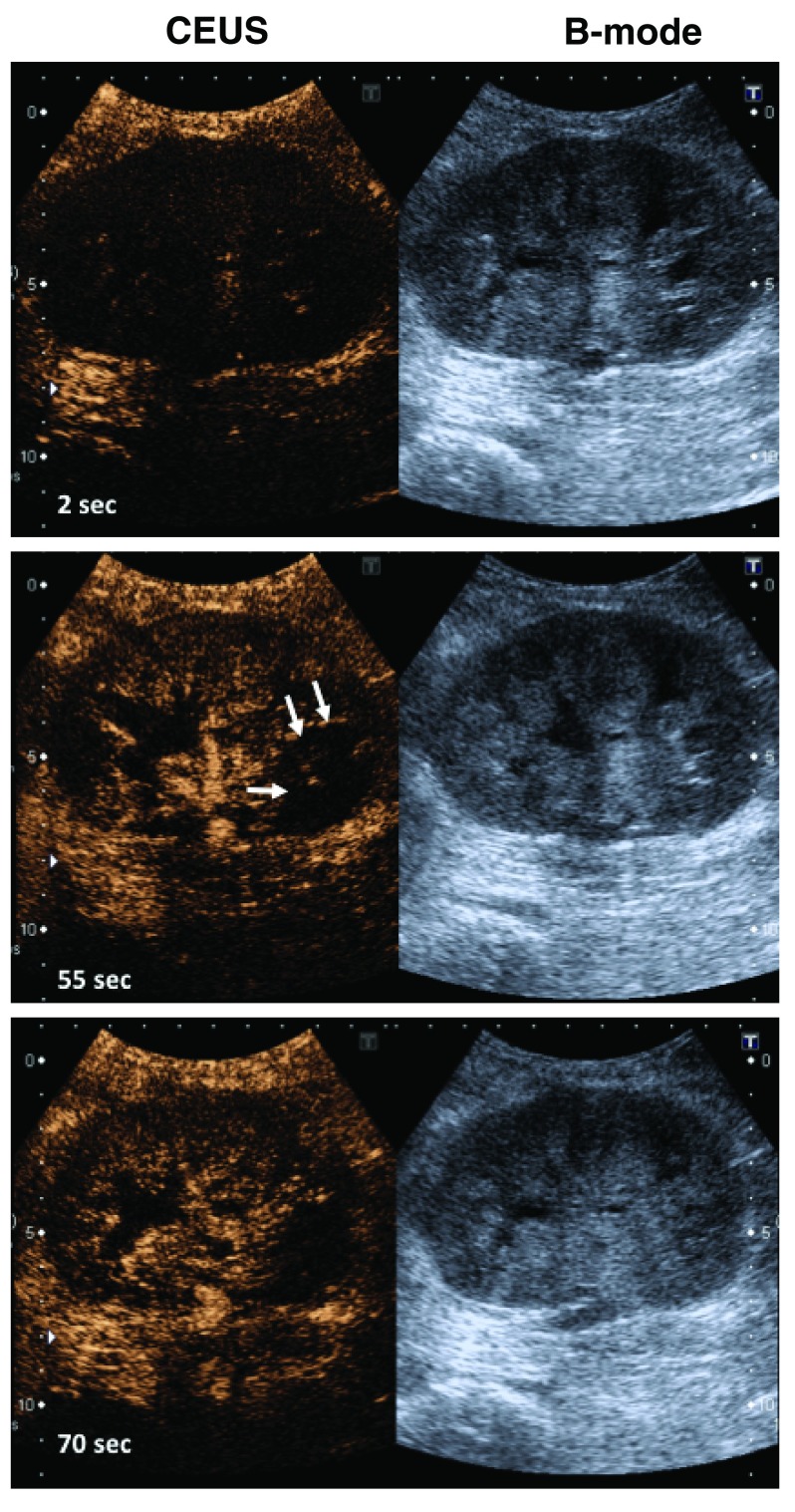
Contrast enhanced ultrasound (CEUS) and B-mode ultrasound of kidney allograft with acute rejection. The left panel shows the enrichment of the microbubbles within seconds in the renal vessels of the allograft of a patient with severe rejection. Areas in the lower pole of the kidney are hypoperfused after microbubble injection, indicating less blood flow to this region (arrows). The right panel corresponds to the B-mode ultrasound. These images are original, unpublished data (F. Gueler).

Microbubbles are synthetic, gas-filled bubbles that can be used as ultrasound contrast agents ^25^. Because of their size (1–4 μm), microbubbles remain intravascular and can be used to directly measure RBF. Microbubbles are generally non-toxic, and no adverse renal effects have been reported, although molecules on their outer shell can be immunogenic. Contrast-enhanced ultrasound (CEUS) is a more accurate method than traditional Doppler measurements for measuring kidney perfusion ^26^. A pilot study in transplant patients with acute rejection also revealed that regional perfusion defects of the allograft can be detected with CEUS ( Figure 4). In addition, CEUS is a valuable and safe method to characterize atypical renal cystic lesions or for follow up on tumor recurrence of renal masses ^27^.

### Oxygen delivery

Oxygen levels within a tissue are a product of both oxygen delivery and oxygen demand. Kidneys are metabolically active organs, and functional measures of tissue oxygenation may reveal physiologic hypoxia, even with preserved RBF. Decreased oxygen levels are probably an important part of the pathogenesis of both acute and chronic kidney injury. Blood oxygen level-dependent (BOLD) MRI is a method that utilizes differences in the magnetic properties of oxygenated and deoxygenated hemoglobin to evaluate tissue oxygen levels. The R2* relaxation rate is inversely related to tissue oxygen levels and can be mapped throughout BOLD MRI of the kidneys ^28, 29^. Kidney oxygenation in patients with CKD and hypertension and healthy controls has been evaluated by BOLD MRI ^30^. Although baseline R2* levels were similar in all three groups of subjects, R2* levels decreased to a greater degree in healthy subjects after the administration of furosemide. This suggests that the relationship between kidney metabolism and sodium transport is abnormal in patients with CKD and hypertension. Focal decrease in R2* levels was also seen in patients with renal arterial stenosis ^31^. Several factors can confound R2* in the kidney, including hydration status, salt intake, tobacco use, and respiratory status, but methods are being developed to help compensate for these factors ^28^.

### Glomerular filtration

Filtration of blood within the kidney is the primary method by which the kidney clears metabolic waste. Serum markers of GFR (e.g. serum creatinine) are commonly measured but often do not accurately reflect reductions in the GFR. Dynamic contrast-enhanced (DCE) MRI is a method in which MRI is used to monitor kidney perfusion and the filtration of GBCAs through the kidney ^32, 33^. Serial images of the kidney are obtained as the contrast is filtered, and the images are used to calculate GFR. Different methods of calculating the GFR from the MRI have been analyzed and compared to radionuclide measurements of GFR in human subjects ^34^. The DCE MRI methods correlated fairly well with radionuclide measurements (correlation coefficient >0.8).

## Imaging of electrolytes and metabolites

### Sodium magnetic resonance imaging

The conventional understanding of sodium handling is that sodium ions are predominantly maintained in the extracellular space through transport out of cells by the Na ^+^–K ^+^ ATPase and that sodium accumulation (or loss) by the body is accompanied by changes in total body water to maintain a relatively constant extracellular osmolarity. However, new MRI methods have expanded our view of sodium homeostasis. Sodium MRI ( ^23^Na MRI) is a method that measures sodium cations instead of ^1^H protons. Although less sensitive than ^1^H MRI, ^23^Na MRI uses specialized equipment and pulse sequences to quantify and localize sodium within tissues ^35^. Increased levels of sodium in a particular tissue can be due to storage in the extracellular space or increased intracellular sodium due to loss of cell integrity ^35^. ^23^Na MRI has been used to measure sodium in various regions of the kidney as well as to measure the accumulation of sodium in other organs.

**Figure 5.**
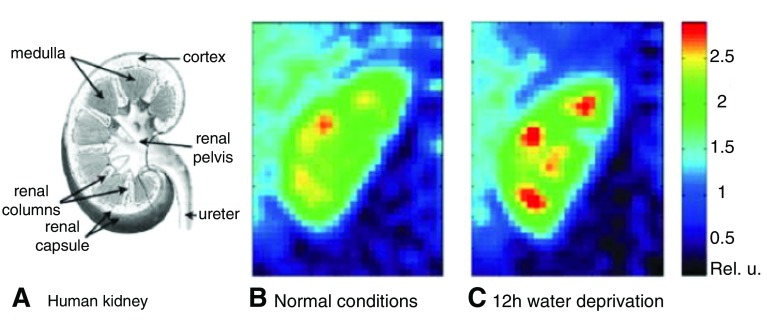
Sodium magnetic resonance imaging (MRI) of the kidney. **A**) A scheme of the human kidney. **B**) Color-coded central coronal slices of sodium images of a human kidney under normal conditions. **C**) Sodium images of the same kidney after 12 hour water deprivation. The sodium gradient increased by 25% after water deprivation. Reproduced with permission from 41.

Within the kidney, reabsorption of water in the distal tubule and collecting duct requires that the extracellular fluid in the renal medulla is hypertonic (~1,200 mOsm/kg in the tip of the medulla). Approximately half of the tonicity of the medulla is due to sodium and the rest is primarily due to urea. The corticomedullary gradient decreases in the injured kidney owing to a reduction in the ability to transport sodium into the medulla, impairing water reabsorption. ^23^Na MRI can measure sodium levels in the renal medulla and determine the corticomedullary sodium gradient ^36, 37^. In humans, the corticomedullary sodium gradient shows wide variation between individuals and does not seem to correlate with other physiologic factors such as age, gender, or body mass index ^36^. Using ^23^Na MRI, investigators have shown that the corticomedullary gradient is lost in models of urinary obstruction, ischemic AKI, and radiation injury, likely reflecting an impaired ability to actively maintain the gradient ^37– 40^. Conversely, the gradient increases in patients with water deprivation ( Figure 5) ^41^.

A key discovery using ^23^Na MRI is that a substantial amount of sodium is deposited in the skin, muscle, and brain ^42^. Detectable levels increase in patients with aldosteronism, which causes renal sodium retention, and decrease after treatment. Surprisingly, interstitial sodium can be stored in these organs without concomitant water, and high local concentrations of sodium in these tissues are not in equilibrium with serum sodium ^43^. Increased tissue storage of sodium is seen in several diseases, including hypertension ^44^ and ESRD ^45^. Interestingly, sodium deposition may be higher in patients with ESRD due to diabetic kidney disease than in patients with ESRD from other causes ^46^. Tissue sodium stores are also higher in patients with AKI than in healthy control subjects ^47^. The correlation of tissue sodium with hypertension and other diseases suggests that these sodium stores may be a marker of cardiovascular risk and/or have a role in disease. Although ^23^Na MRI is not yet available for clinical use, in the future this method may be useful for measuring total body sodium levels, examining cell integrity, and monitoring therapies intended to remove sodium from the body.

### Hyperpolarized magnetic resonance imaging

Hyperpolarization is a method whereby energy is transferred to the nuclear spin of a compound that contains carbon-13 ( ^13^C) ^48^. Hyperpolarized compounds have a very high signal by MRI, although the increased signal is short-lived and the hyperpolarization needs to be done near where the imaging will be performed. Hyperpolarized probes can be injected into a subject, and the compound and its metabolites can then be traced for several minutes by MRI. A study using hyperpolarized ^13^C pyruvate showed that metabolism of pyruvate to lactate is altered in models of diabetic nephropathy ^49^. ^13^C urea can also be polarized and its localization in the kidney imaged by MRI ^50^. Renal uptake of hyperpolarized urea demonstrated altered urea transport in rats with experimental diabetic nephropathy ^48^ or with IRI ^51^. MRI of hyperpolarized metabolites could provide a useful method of monitoring transport and metabolic functions in the kidney, but the short duration of the probes is a barrier to the clinical application of these methods.

## Inflammation

One of the most common reasons to perform a kidney biopsy is to diagnose glomerulonephritis or to assess the disease activity of patients with known disease. The detection of glomerular inflammation, or “active” disease, is frequently used to make treatment decisions. Furthermore, repeat biopsies may be necessary to diagnose disease recurrence or to assess whether or not a patient is responding to treatment. Glomerular inflammation is often patchy, however. Kidney biopsies may not accurately represent inflammation throughout the kidneys, particularly if the number of glomeruli is small ^52^. Kidney allograft biopsies are still the gold standard to grade transplant rejection and to distinguish other forms of allograft injury. Several new imaging methods and probes have been developed to detect cellular or molecular markers of kidney inflammation ^53^.

Inflammation causes vascular leakage and edema formation in tissues. This can be visualized by T1 and T2 mapping of MRI as a measure of tissue water content ^54^. The T1 relaxation time, a measure of how fast the nuclear spin magnetization returns to its equilibrium state after a RF pulse in the MRI scanner, is a key source of soft tissue contrast in MRI ^55^. Changes in T1 relaxation time reflect edema, infarction, and scarring ^56^. RBF, as assessed by ASL, is also reduced in patients with kidney inflammation. For example, RBF is reduced in patients with lupus nephritis compared to healthy controls ^57^. Because these MRI measurements are also reduced in patients with CKD, however, the interpretation of the results requires consideration of the context and timing in disease progression.

### Ultrasound probes for detecting inflammation

Standard ultrasound can detect abscesses and gross tissue changes, but it cannot accurately detect kidney inflammation. Conjugation of targeting proteins to the surface of microbubbles can direct them to endothelial markers of inflammation, such as adhesion molecules, providing a signal of tissue inflammation. A preclinical study used microbubbles targeted to P-selectin and vascular cell adhesion molecule 1 (VCAM-1) to test whether they could be used to detect kidney inflammation after ischemia reperfusion ^58^. A strong signal was seen four hours after the induction of ischemia/reperfusion using both probes. Microbubbles targeted to other molecules have also been used to detect renal inflammation, including the detection of T cells in a model of allograft rejection ^59^, and activated neutrophils in a model of ischemic AKI ^60^. These methods may be useful for detecting markers of allograft rejection that currently require a tissue biopsy to identify them.

### Magnetic resonance imaging probes for detecting inflammation

MRI probes have also been used to detect markers of inflammation within the kidney. Superparamagnetic iron oxide (SPIO) causes a darkening of T2-weighted MRI, and SPIO can be used as a contrast agent ^61^. SPIO that are 20–30 nm in diameter are phagocytosed by macrophages, thereby accumulating in tissues infiltrated by macrophages. They can therefore be used as a molecular imaging probe for macrophages within the kidney. Using untargeted SPIO, tissue macrophages were detected in animal models of focal segmental glomerulosclerosis, acute glomerulonephritis, and ischemic AKI ^62– 64^. In a pilot study of human patients, signal was seen in patients with acute rejection of a renal transplant, glomerulonephritis, or ischemic AKI ^65^. Furthermore, the localization of the signal was in the medulla of patients with AKI, and in the cortex of patients with the other causes of inflammation, and the change in MRI signal in the kidney after SPIO injection correlated with infiltration of the kidney by macrophages.

Similar to microbubbles, SPIO nanoparticles can be conjugated with targeting molecules that cause them to accumulate in tissues expressing the target ligands. SPIO targeted to tissue-bound complement C3 fragments caused negative enhancement of the kidneys in a model of lupus nephritis ^66^. A subsequent study showed that the magnitude of the change in MRI signal after injection of the targeted SPIO correlated with disease severity ^67^. Kidney biopsies from patients with lupus nephritis are routinely immunostained for C3 deposits, and this imaging method holds promise as a non-invasive way of monitoring this marker of kidney inflammation.

Kidney inflammation is also associated with metabolic changes. Chemical exchange saturation transfer is an MRI method that can be used to measure metabolites within a tissue ^68^. Using this technique to measure glucose content (GlucoCEST) can be employed as a readout of metabolism. GlucoCEST readings in different areas of the kidney were able to distinguish acute rejection from other forms of injury in a rodent transplant model ^69^. Interestingly, the readings did not correlate with renal perfusion in a model of ischemic injury.

### Nuclear medicine probes for detecting inflammation

**Figure 6.**
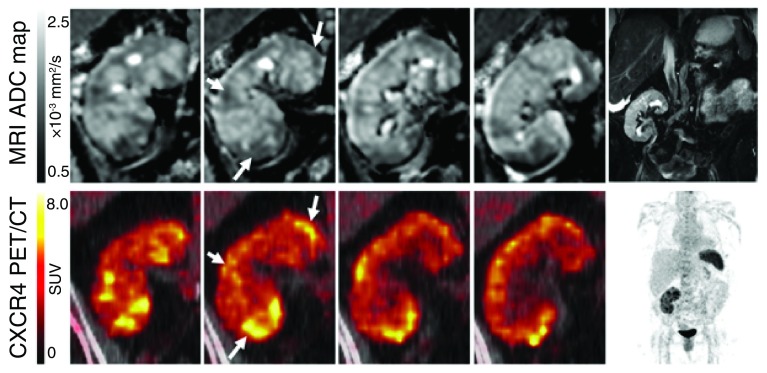
Magnetic resonance imaging (MRI) and CXCR4-positron emission tomography (PET) imaging of acute renal allograft infection. Diffusion-weighted MRI and PET imaging with a specific CXCR4 ligand ( ^68^Ga-pentixafor) were used to image the kidney of a patient with recurrent urinary tract infection after kidney transplantation. The upper row shows apparent diffusion coefficient (ADC) maps of the kidney (top, frontal views from posterior to anterior), and the bottom row shows ^68^Ga-pentixafor PET imaging. Areas of reduced ADC corresponded to foci with upregulated CXCR4 expression (arrows). T2-weighted MRI and maximum-intensity-projection PET are shown for anatomic orientation. The kidney allograft is located in the right lower abdomen. The spleen displays high CXCR4 expression due to high content of leukocytes. Reproduced with permission from Derlin *et al.* ^70^. SUV, standardized uptake value.

Positron emission tomography (PET) can detect specific molecules with high sensitivity and has been used to quantify inflammatory markers in target tissues. Chemokine receptor CXCR4-targeted PET can detect leukocyte infiltrates ( Figure 6). A recent study integrated CXCR4-targeted PET with MRI as a method of localizing infections in transplant patients with complicated urinary tract infections (UTIs) ^70^. In this setting, it can be difficult to determine whether the primary site of the UTI is the transplanted kidney or the heavily damaged native kidneys. This study examined patients who were already scheduled for native nephrectomy. In some patients, the CXCR4 imaging revealed severe allograft pyelonephritis rather than infection of the native kidney. The knowledge of the primary source of infection and the extension of the local inflammation can help to optimize the therapeutic approach for the individual patient, and, in some cases, it can even prevent the unnecessary removal of unaffected kidneys.

## Fibrosis

Kidney fibrosis is a hallmark of most forms of tissue injury. It is the end result of essentially all kidney diseases, including genetic diseases, acute or chronic ischemia, infection and inflammation, ionizing radiation, and obstructive nephropathy. Glomerulosclerosis and tubulointerstitial fibrosis seen on kidney biopsy are generally interpreted as irreversible lesions, although drugs to reverse fibrosis are in development ^71^. The detection and quantification of fibrosis is clinically very important, as tubulointerstitial fibrosis is a strong predictor of progressive renal failure and the detection of extensive fibrosis may prevent clinicians from using treatments that are unlikely to be effective ^72^.

Standard B-mode ultrasound is commonly used to look for kidney fibrosis. Fibrotic changes increase the echogenicity (brightness) of the kidney cortex and decrease the thickness of the cortex, and the renal contour can appear irregular. Irreversible kidney damage is also associated with decreased kidney size (usually estimated by the length) ^1^. However, these findings are not sensitive or specific for fibrosis, and they do not accurately reflect changes to the composition of the kidney and accumulation of extracellular matrix. Given the importance of detecting and quantifying kidney fibrosis, several new imaging probes and methods are being developed for this purpose.

Elastography reports on the stiffness of tissues by measuring deformation of the tissue to applied pressure, and several ultrasound techniques have been adapted to measure stiffness of the kidney. An impulse is applied to the kidney in order to generate shear waves, and the speed of the shear waves (shear wave velocity [SWV]) is then measured. Stiffness of the tissue would be expected to increase the SWV, and this was observed in a rat model of kidney disease ^73^. Surprisingly, in two studies of patients with CKD, the SWV decreased in patients with more severe disease ^74, 75^. Two other studies examined whether elastography could identify kidney fibrosis. In a study of patients with diabetic nephropathy, the SWV values did correlate with the degree of kidney disease ^76^. A study of renal transplant recipients, on the other hand, did not find any correlation between renal elastography and kidney fibrosis ^77^. It is possible that these discrepant results are explained by the effects of other factors, such as the surrounding tissues and urine in the collecting system, on the shear wave imaging readings ^78, 79^.

Acoustic radiation force impulse imaging is an elastography-based method that can be used to create a map of tissue stiffness throughout an organ ^80^. Values in the kidneys of patients with CKD are lower than those in healthy controls, and the results correlate with the degree of kidney dysfunction ^81^. In another study, this method distinguished patients with diabetic nephropathy from healthy control kidneys ^76^.

Several MRI methods can also be used to evaluate renal fibrosis, and efforts are underway to standardize these techniques ^82^. Similar to ultrasound elastography, MR elastography can be used to measure kidney stiffness as an indicator of fibrosis. In MR elastography, MRI is used to measure displacement of the kidney in response to compression or mechanical vibration. MR elastography was initially developed to assess hepatic fibrosis, but more recently it has been adapted as a method for measuring kidney fibrosis. As with ultrasound elastography, the results are affected by factors other than fibrosis, including tissues adjacent to the kidney, RBF, and urine in the renal pelvis ^79, 83^. MR elastography correlated with tissue fibrosis in a pig model of ischemic nephropathy ^84^. A small study in kidney transplant recipients also showed that MR elastography may correlate with kidney fibrosis on biopsy, although the number of patients was too small to be conclusive ^85^.

Diffusion weighted imaging (DWI) is an MRI method for studying the movement of water molecules in a tissue. Different methods can be used to distinguish directional flow (such as the flow of blood or urine) and random diffusion ^86, 87^. In fibrotic tissues, the water molecules become more constrained, decreasing a metric called the apparent diffusion coefficient (ADC). In a mouse model of progressive kidney fibrosis, the ADC decreased in proportion to histologic fibrosis ^88^. In humans, ADC values were shown to correlate with decreased kidney function ^89, 90^.

A related MRI method called diffusion tensor imaging (DTI) also measures the mobility of water molecules, but it incorporates analysis of the direction in which water molecules can move. Changes in DTI have been associated with histologic damage on kidney biopsy, including tissue fibrosis ^91– 93^. Because DWI and DTI are based on the motion of water molecules in tissue, however, they can be affected by factors other than fibrosis, including renal perfusion, tubular dilatation, or other changes to tissue architecture ^94^. Renal fibrosis is also associated with a loss of capillaries, for example, reducing blood flow through the parenchyma, but RBF can also be affected by heart failure or hydration status. Furthermore, the values for these imaging measurements in healthy subjects overlap with those for patients with disease, limiting the utility of these methods in individual patients ^90^.

## Lipid content

In addition to inflammation and fibrosis, pathologic processes can also change the composition of the kidneys in other ways. For example, obesity and diabetes are associated with lipid accumulation and fatty changes in the kidneys. Several MRI methods have been developed to detect these changes and have been tested in patients with metabolic syndrome or diabetes ^95– 98^. Although the role for lipid accumulation as either a marker or a cause of kidney disease is incompletely understood, methods for detecting these changes will allow investigators to determine whether they are of prognostic importance.

## Conclusions

Many promising new radiologic methods and tools that may help in the detection of kidney disease are currently in development. These methods can detect with high resolution alterations in the structure of the kidney as well as readouts of blood flow or specific kidney functions. New molecular imaging methods can also detect markers of inflammation and fibrosis within the kidneys. Although these methods have not yet entered clinical practice, many of them can be performed using equipment that is widely available. MRI and PET-CT imaging methods remain quite costly, however, and are currently not fully reimbursed by insurance companies. The new methods are therefore still mostly used within clinical studies. Nevertheless, better imaging methods hold great promise for diagnosing diseases earlier and for safely monitoring the responses of patients who undergo treatment.
